# A prognostic model and immune regulation analysis of uterine corpus endometrial carcinoma based on cellular senescence

**DOI:** 10.3389/fonc.2022.1054564

**Published:** 2022-12-08

**Authors:** Lulu Gao, Xiangdong Wang, Xuehai Wang, Fengxu Wang, Juan Tang, Jinfeng Ji

**Affiliations:** ^1^ Department of Obstetrics and Gynecology, Nantong Maternal and Child Health Hospital Affiliated to Nantong University, Nantong, China; ^2^ Department of Integrated Traditional Chinese and Western Internal Medicine, Nantong Tumor Hospital, Affiliated Tumor Hospital of Nantong University, Nantong, China; ^3^ Department of Occupational Medicine and Environmental Toxicology, Nantong Key Laboratory of Environmental Toxicology, School of Public Health, Nantong University, Nantong, China

**Keywords:** UCEC, cellular senescence, bioinformatics, prognosis, multi-omics analysis

## Abstract

**Background:**

This study aimed to explore the clinical significance of cellular senescence in uterine corpus endometrial carcinoma (UCEC).

**Methods:**

Cluster analysis was performed on GEO data and TCGA data based on cellular senescence related genes, and then performed subtype analysis on differentially expressed genes between subtypes. The prognostic model was constructed using Lasso regression. Survival analysis, microenvironment analysis, immune analysis, mutation analysis, and drug susceptibility analysis were performed to evaluate the practical relevance. Ultimately, a clinical nomogram was constructed and cellular senescence-related genes expression was investigated by qRT-PCR.

**Results:**

We ultimately identified two subtypes. The prognostic model divides patients into high-risk and low-risk groups. There were notable discrepancies in prognosis, tumor microenvironment, immunity, and mutation between the two subtypes and groups. There was a notable connection between drug-sensitive and risk scores. The nomogram has good calibration with AUC values between 0.75-0.8. In addition, cellular senescence-related genes expression was investigated qRT-PCR.

**Conclusion:**

Our model and nomogram may effectively forecast patient prognosis and serve as a reference for patient management.

## Introduction

Uterine corpus endometrial carcinoma (UCEC) is one of the three major gynecological malignancies, second only to cervical cancer in incidence (1, 2). Hypertension, diabetes, obesity, infertility, and family history are risk factors for UCEC (3). However, because of the scarcity of effective timely detection of UCEC, many patients have progressed to advanced stages by the time they are diagnosed (4). At the same time, the poor prognosis for patients who develop metastases despite treatment is now a pressing issue (5). Treatment options other than first-line chemotherapy drugs remain limited (6). Studies show that the treatment and prognosis of patients can be assessed through predictive models and biomarkers (7). However, there are no credible biomarkers to assess the outcome for UCEC.

Cellular senescence is the central process of aging, bringing the cell cycle to a permanent standstill (8). Cellular senescence can promote repair and prevent tumorigenesis. Meanwhile, some degenerative diseases and cancers are associated with abnormal accumulation of senescent cells (9, 10). Senescent tumor cells can modulate the tumor microenvironment (TME), transform surrounding unsenescent cells into senescent cells, and recruit and activate immune cells to produce anti-tumor and pro-tumor effects (8, 9). Cellular senescence is capable of limiting tumor growth progression and is considered a potential therapeutic target (11). Adriamycin and bleomycin can induce senescence and thus exert anti-tumor effects. Therefore, studying the effects of cellular senescence in tumors can help develop new approaches to tumor therapy (12). However, the role of cellular senescence in UCEC and the relationship with UCEC prognosis remains unclear.

## Materials and methods

### Data collection

From TCGA and GEO databases, the gene expression and clinical data of UCEC were downloaded. The GEO cohort GSE119041 and TCGA cohort were acquired (13). Among them, patients in the integrated cohort of the TCGA cohort and the GEO cohort were randomly divided into training cohort and testing cohort at the ratio of 1:1, the integrated cohort was also defined as validation cohort. We normalized the expression of the genes by using “ComBat” algorithm from the “sva” package (14). Patients with inadequate clinical data and survival information were eliminated.

### The clustering analysis

We collected 307 cellular senescence related genes from the previous study (15). Full details of these genes were shown in **Table S1**. The “ConsensusClusterPlus” package was used to perform consistent unsupervised cluster analysis to classify patients into different subtypes. We screened out clusters with high intra-type correlation and low inter-type correlation for subsequent analysis (16).

### Multi-omics analysis of UCEC subtypes based on senescence genes

First, to validate the categorization of patient subtypes, we used principal component analysis (PCA). We investigated the link with the subtypes and patient clinical characteristics. We then performed a survival analysis using the “survival” package to draw Kaplan–Meier curves to assess differences in survival between subtypes. Next, we explored the differences in the TME between different subtypes. Violin plots were used to show the distribution of TME scores for each sample across subtypes. a score of 22 immune cells was obtained by the CIBERSORT method (17). To measure the amount of immune cell infiltration, the single sample gene set enrichment analysis (ssGSEA) technique was utilized (18). Finally, we explored differences in PD-L1 and PD-L2 expression among different subtypes.

### Enrichment analysis

Using the “clusterProfiler” software package, we performed Gene Ontology (GO) analysis to identify functions for these genes, and the Kyoto Encyclopedia of Genes and Genomes (KEGG) analysis to identify enriched pathways for these genes (19). We retained analysis results with p-values less than 0.05 and displayed them in bar graphs.

### Difference analysis

Based on gene expression between the two subtypes, we screened for genes that differed between the two subtypes (20). In addition, we analyzed the pathways that differed between the two subtypes by means of KEGG enrichment analysis.

### The differential genes clustering analysis and multi-omics analysis

First, we used the same method as above for cluster analysis. Then, we explored the association of this subtype with clinical factors and performed survival analysis. Besides, we performed TMB analysis and checkpoint analysis of PD-L1 and PD-L2.

### Model construction and evaluation

In the training cohort, we performed the least absolute and selection operator (LASSO) regression analysis to select cellular senescence related genes to connect to the prognosis. The model’s predictive performance was tested using test and validation cohorts. Based on the median risk score, we classified the patients into two groups: high-risk and low-risk. Between the two groups, we investigated variations in clinical features and patient outcomes. The time-dependent receiver operating characteristic (ROC) curve was utilized to assess the model’s accuracy. Besides, univariate and multivariate cox analyses were also performed (21).

### Multi-omics analysis for the model

First, the link between risk scores and clinical factors was investigated. We then explored the TME based on the model. One-class logistic regression (OCLR) machine-learning algorithm was used to quantify the stemness of tumor samples by calculating cancer stem cell indices (22). Pearson analysis was used to reveal the correlation of risk score and RNAss. Between the two groups, the GSEA analysis was carried out to evaluate variations in enriched pathways. Besides, we also performed immune microenvironment (IME) analysis. We immunotyped the patients and investigated the association with both risk score and immunotyping to learn more about the based on risk score and immunity.

Studies showed that tumor mutational burden (TMB) correlates with IME (23). Therefore, we calculated TMB for each sample by somatic mutation profiles and investigated the link between risk score and TMB. Based on the median TMB, we separated patients into high-TMB and low-TMB groups and performed survival analysis. In addition, we combined TMB with risk scores for survival analysis. Besides, we analyzed the relationship among riskscores and microsatellite instability (MSI) and immunophenoscore (IPS).

The “PRROPHOPIC” pack includes hundreds of medicines (24). From it, we calculated the half inhibitory concentration (IC50) value of the drug and screened out the drugs with significant differences in the two risk groups.

### Nomogram construction and evaluation

We created a nomogram using the riskscores and clinical data. The nomogram’s accuracy was assessed using the C-index, ROC curve, and calibration curve.

### Quantitative RT-PCR

A total of 12 UCEC tissues from patients in the Nantong Maternal and Child Health Hospital Affiliated to Nantong University were paired with normal tissues. The Ethics Committee of the Nantong Maternal and Child Health Hospital Affiliated to Nantong University approved the study. All patients signed the informed consent form. Use TRIZOL reagent (Thermo Fisher Scientific, USA) to separate total RNA from the sample, then use Revert Aid first strand cDNA synthesis kit (Thermo Fisher Scientific, USA) to reverse transcribe it into cDNA, and use SYBR Green PCR kit (Takara, Tokyo, Japan) for real-time quantitative PCR (qRT-PCR) analysis. GAPDH was used to regulate the relative expression of genes. The sequence is listed in **Supplementary Table S3**.

## Results

### Establishment and assessment of senescence subtypes

**Figure 1:**
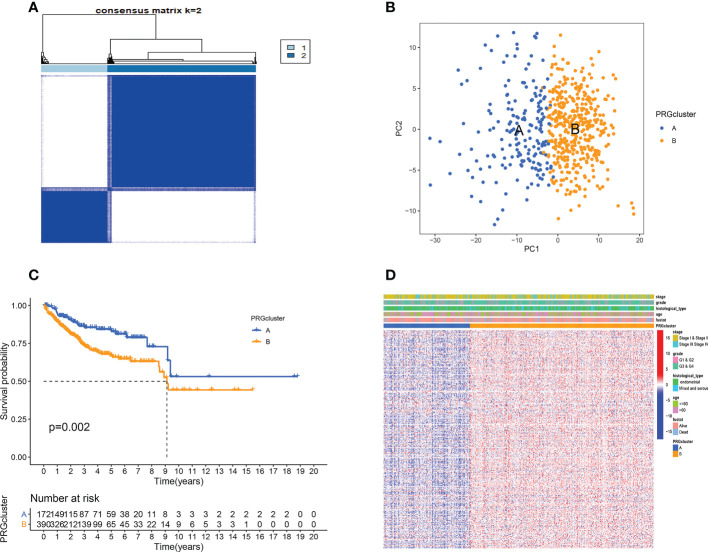
Cellular senescence subtypes and clinical assessment. **(A)** Two subtypes and their associated regions. **(B)** PCA analysis. There are significant differences between the two subtypes. **(C)** Survival analysis. Subtype B has a poorer prognosis. **(D)** There were no differences in clinical factors between the two subtypes.

We included 593 patients from both TCGA and GEO cohorts in our study for further analysis. Based on cellular senescence related gene expression, we classified patients using a consensus clustering approach(**Figure S1**). The results of the analysis show that k=2 is the optimal number of groups (**Figure 1A**). We then divided them into subtype A and subtype B based on the above results. PCA analysis indicated that subtypes A and B successfully distinguished patients(**Figure 1B**). Survival analysis incidated that our subtype successfully stratified the survival of patients, and the survival time of subtype A was longer (**Figure 1C**). However, after comparing the clinical factors of the patients, we found no difference in the expression of pyroptotic genes with age, stage, grade, survival status, and histological type (**Figure 1D**).

### Multi-omics analysis of different senescence subtypes

**Figure 2:**
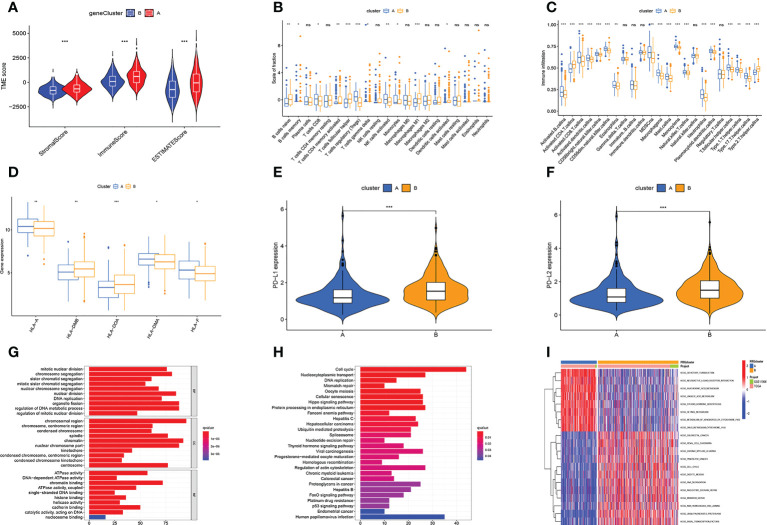
Multi-omics analysis based on senescence cluster. **(A)** TME analysis. Subtype A has a higher TME score. **(B, C)** Differences in immune cell infiltration levels. **(D)** Differences in HLA-related gene expression levels. **(E, F)** The PD-L1 and PD-L2 genes of subtype B are highly expressed. **(G, H)** The GO **(G)** and KEGG **(H)** enrichment analysis. **(I)** Differential KEGG enriched pathways between the two subtypes. Adjusted p-values were shown as ns, not significant; *p < 0.05; **p < 0.01; ***p < 0.001.

TME plays a key role in tumorigenesis and progression. Therefore, we first analyzed the TME. Violin plots showed significant differences in stromal, immune, and ESTIMATE scores between the two subtypes (**Figure 2A**). We further analyzed the immune-related functions and infiltration of immune cells of two subtypes based on the above results. A subtype had higher infiltration levels of NK cells activated, T cells regulatory, and T cells CD8, while B cells naive, T cells follicular helper, and Macrophages M1 had greater levels of infiltration in the B subtype (**Figure 2B**). ssGSEA analysis further confirmed that immune cell infiltration levels differed significantly between the two subtypes (**Figure 2C**). Besides, the expression of HLA-A, HLA-DMA, and HLA-F was higher in subtype A, while the expression of HLA-DMB and HLA-DOA in subtype B was higher (**Figure 2D**). The results of the checkpoint analysis indicated B subtype showed greater levels of PD-L1 and PD-L2 expression (**Figures 2E, F**).

We also analyzed gene function and enriched pathways. GO enrichment analysis revealed these genes were primarily associated with cell mitosis, metabolism of genetic material, and ATP metabolism (**Figure 2G**). KEGG enrichment analysis revealed these genes were primarily associated with cell cycle, protein processing, transport, and DNA replication (**Figure 2H**). Besides, it also revealed subtype A was substantially more concentrated in lipid metabolism, and subtype B had considerable cell cycle, cell division, and tumor enrichment (**Figure 2I**).

### Differential genes subtypes

Through differential analysis, we identified 1219 differential genes. Based on these genes, we used the same cohort and method to further subtype the patients (**Figure S2A**). We found dividing patients into two subtypes (A and B) was optimal (**Figure S2B**). Besides, the survival time of the two subtypes was significantly different (**Figure S2C**). However, the heatmap showed no differences in clinical factors between the two subtypes (**Figure S2D**).

**Figure 3:**
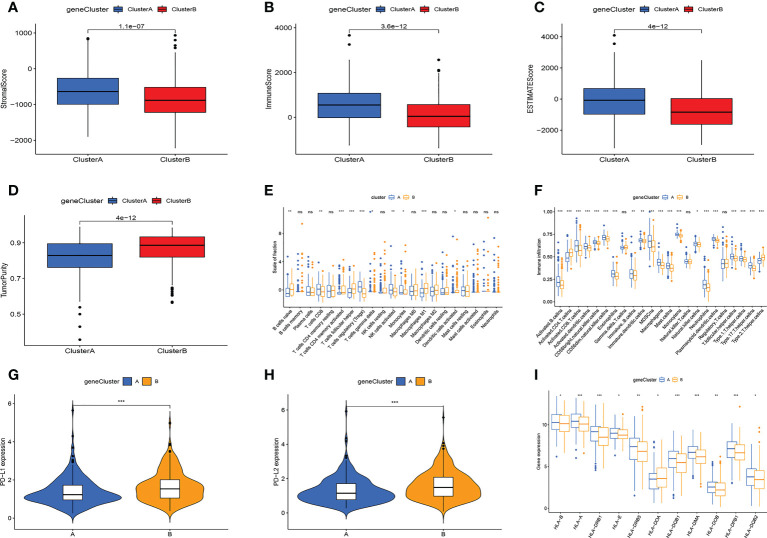
Multi-omics analysis based on differential genes cluster. **(A-D)** TME analysis. Subtype A has higher TME scores and subtype B has higher tumor purity. **(E, F)** The amount of immune cell infiltration differed significantly. **(G)** PD-L1, PD-L2 expression levels are higher in subtype **(B-H)** The A subtype has higher expression levels of HLA-related genes. Adjusted p-values were shown as ns, not significant; *p < 0.05; **p < 0.01; ***p < 0.001.

Then, we performed TME analysis. The results showed that subtype A had higher stromalscore, immunescore, and estimatescore, while subtype B had higher tumorpurity (**Figures 3A–D**). In addition, the A subtype of NK cells activated, T cells regulatory (Tregs), and T cells CD8 have a higher degree of infiltration, and the B subtype of Macrophages M1, T cells follicular helper, and B cells naive have a higher degree of infiltration (**Figure 3E**). The results of ssGSEA analysis further confirmed that immune cell infiltration differed significantly between the two subtypes (**Figure 3F**). At the same time, the PD-L1 and PD-L2 genes of subtype B are highly expressed (**Figures 3G, H**). **Figure 3I** showed that the expression of HLA-related genes of the two subtypes was significantly different. This is basically consistent with the analysis of cellular senescence subtypes.

### Model construction and evaluation

**Figure 4:**
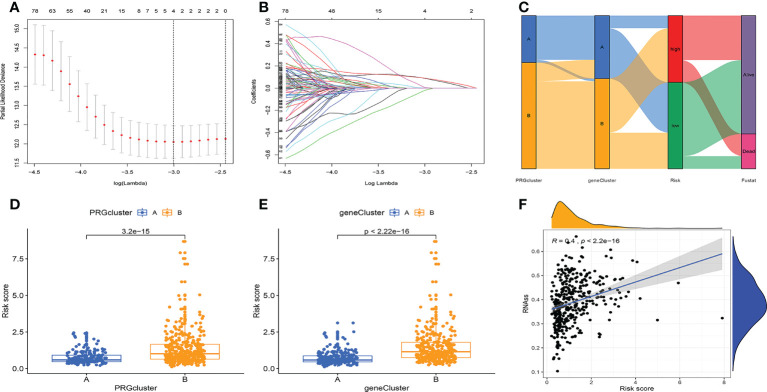
Prognostic model construction. **(A, B)** LASSO regression analysis. 4 genes were screened to build a prognostic model. **(C)** Distribution of different subtypes, risk groups, and survival outcomes. **(D, E)** Distribution of risk scores for different subtypes. **(F)** RNAss values are positively correlated with risk scores.

After LASSO analysis, a total of 4 genes were screened (**Figures 4A, B**). The model’s calculating formula was as follows: riskscore = BZW2*0.44481118 - NRIP1*0.38695576 + ARHGAP29*0.22408622 + SIX1*0.18719355. Based on the median risksocre in the training cohorts, patients in the three cohorts were separated into high- and low-risk groups. **Figure 4C** showed the distribution of patients grouped by two cellular senescence subtypes, two differential gene subtypes, high and low-risk groups, and survival status. We also observed that both the cellular senescence subtype and the differential gene subtype had a higher risk score for the B subtype (**Figures 4D, E**). **Figure 4F** shows that RNAss values are positively correlated with risk scores. Furthermore, the risk score was linked to patient’s clinical factors. The higher risk score, the more advanced and poorly differentiated tumors, and the greater the likelihood of death (**Figures S3A–D**). We also found a lower risk score for tumors originating from endometrial tissue and a higher risk for mixed and serous tissue (**Figure S3E**).

We then analyzed the relationship of the model to patient survival. Patients were separated into high-risk and low-risk groups based on the median (**Figures S4A–C**). At the same time, the number of patient deaths was proportional to the risk score (**Figures S4D–F**). Furthermore, in the high-risk group, BZW2, ARHGAP29, and SIX1 were overexpressed, whereas NRIP1 was overexpressed in the low-risk group (**Figures S4G–I**).

**Figure 5:**
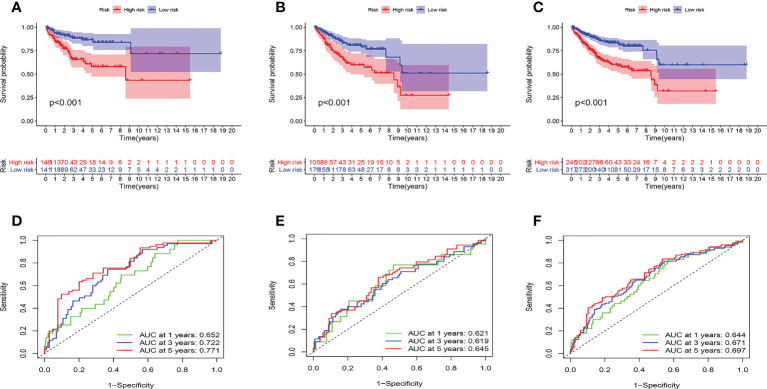
Prognostic model evaluation. **(A-C)** survival analysis. the high-risk group had a worse prognosis in training **(A)**, test **(B)**, and validation **(C)** cohorts. **(D-F)** ROC curves. The AUC value of the model is basically between 0.6 and 0.7 in three cohorts.

Then, we evaluated the accuracy of the model. The high-risk group had the worst prognosis among the three groups (**Figures 5A–C**). **Figures S4A–H** showed the results of survival analysis for clinical factors. The AUC of the training cohort at 1, 3, and 5 years was 0.652, 0.722, and 0.771, respectively (**Figure 5D**). The AUC of the test cohort at 1, 3, and 5 years was 0.621, 0.619, and 0.645, respectively (**Figure 5E**). The AUC of the validation cohort at 1, 3, and 5 years was 0.644, 0.671, and 0.697, respectively (**Figure 5F**).

### Independent prognostic analysis

For independent prognostic analysis, univariate and multivariate COX regression models were utilized. The results of the univariate COX analysis are as follows (**Table S1**). In the training cohort, histological type, stage, riskscore were independent prognostic factors. The grade was also an independent prognostic factor in the testing cohort and validation cohort. In the three cohorts, multivariate COX analysis demonstrated riskscore and stage were independent predictive variables (**Table S2**).

### The model’s multi-omics analysis

**Figure 6:**
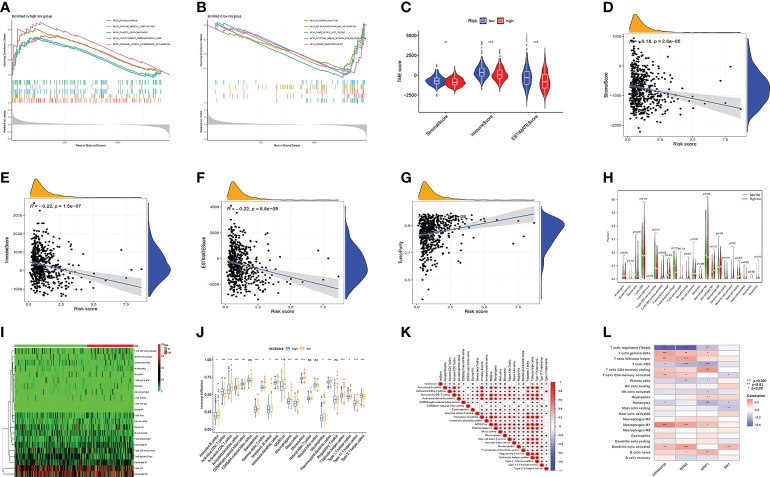
Model multi-omics analysis. **(A, B)** GSEA enrichment analysis. **(C-F)** TME analysis. the low-risk group had higher StromalScore, ImmuneScore, and ESTIMATEScore. Risk Score is inversely proportional to StromalScore, ImmuneScore, and ESTIMATEScore, and proportional to TumorPurity. **(G-I)** The amount of immune cell infiltration differed significantly. **(J)** Immune cell infiltration and risk score were linked. **(K)** immune cells and Risk score were linked. **(L)** immune cells and model genes were linked. Adjusted p-values were shown as ns, not significant; *p < 0.05; **p < 0.01; ***p < 0.001.

First, GSEA analysis revealed the high-risk group was mostly associated with cardio-renal diseases (**Figure 6A**). The low-risk group was mostly associated with immunity and rejection (**Figure 6B**). Then, we analyzed the relationship between the TME and the model. StromalScore, ImmuneScore, and ESTIMATEScore were greater in low-risk group (**Figure 6C**). In addition, riskScore is inversely proportional to StromalScore, ImmuneScore, and ESTIMATEScore, and proportional to TumorPurity (**Figures 6D–G**). **Figure S5A** illustrated the distribution of immune cell in two groups per patient. We then investigated the model’s connection to immune cell infiltration. Besides, T cells CD4 memory activated, T cells follicular helper, T cells regulatory, NK cells resting, Macrophages M1, and Dendritic cells activated were distinct in the two groups (**Figures 6H, I**). SsGSEA analysis also confirmed that in the high-risk group, most immune cells had higher infiltration levels (**Figure 6J**). The risk score was significantly associated with immune cells, model genes (**Figures 6K, L**). We found that T cell regulatory were negatively correlated with riskscore, and all the rest of cells had a positive correlation to risk score. (**Figures S5B–F**). **Figure 6K** showed the relationship between model genes and immune cells. Then, we divided patients into four subtypes based on their immunity (**Figure S5G**). Different types of immune infiltration correspond to tumor promotion and tumor inhibition, including C1 (wound healing), C2 (INF-g dominance), C3 (inflammation) and C4 (lymphocyte depleted) (25). The risk score for the C2 subtype was the greatest, while the risk score for the C3 subtype was the lowest (**Figure S5H**). In addition, significant variations between the two groups were also seen in the expression of immunological checkpoint genes (**Figure S5I**). Among them, CTLA4, PDCD1LG2, and PDCD1 were most associated with risk scores (**Figure S5J**). Risk scores were inversely correlated with PDCD1LG2, CTLA4, and PDCD1, and favorably correlated with PDCD1LG2 (**Figures S5K–M**).

**Figure 7:**
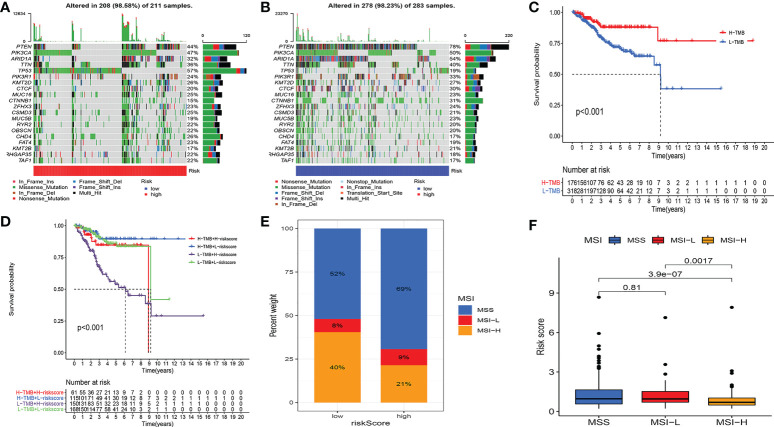
Multi-omics analysis of the model. **(A, B)** Gene mutation frequencies in both groups. **(C, D)** Survival analysis. H-TMB has a better prognosis. **(E, F)** MSI analysis. The low MSI accounted for the least, and the high MSI group had the lowest risk score.

**Figure 8:**
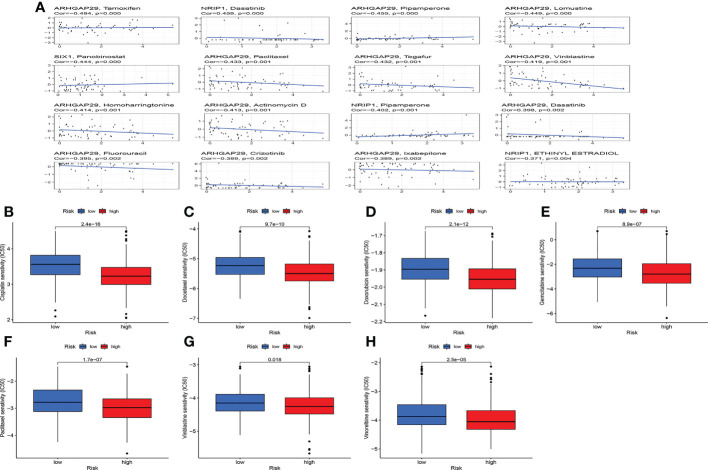
Drug sensitivity analysis. **(A)** Relationship between model genes and sensitive drugs. **(B-H)** Sensitive drugs in high-risk group.

Studies have demonstrated that TMB can serve as an important component of composite predictors to guide tumor immunotherapy (26). We found that the three genes with the greatest mutation probability in the high-risk group were TP53, PIK3CA, and PTEN, while the three genes with the highest mutation probability in the low-risk group were PTEN, ARID1A, and PIK3CA (**Figures 7A, B**). We then performed survival analysis. The prognosis of patients with high-TMB scores and high risk score was greater (**Figures 7C, D**). The research by Ganesh et al. illustrated MSI is closely related to the sensitivity to immunotherapy (27). The low MSI accounted for the least, and the high MSI group had the lowest risk score (**Figures 7E, F**). To further guide the patient’s treatment, we performed a drug sensitivity analysis. First, we screened out drugs related to model genes, including Tamoxifen, Dasatinib, Panobinostat, etc (**Figure 8A**). Next, we further screened drugs sensitive to the high-risk group, including Gemcitabine, Doxorubicin, Docetaxel, Cisplatin, Vinorelbine, Paclitaxel, Vinblastine (**Figures 8B–H**).

### Nomogram construction and validation

**Figure 9:**
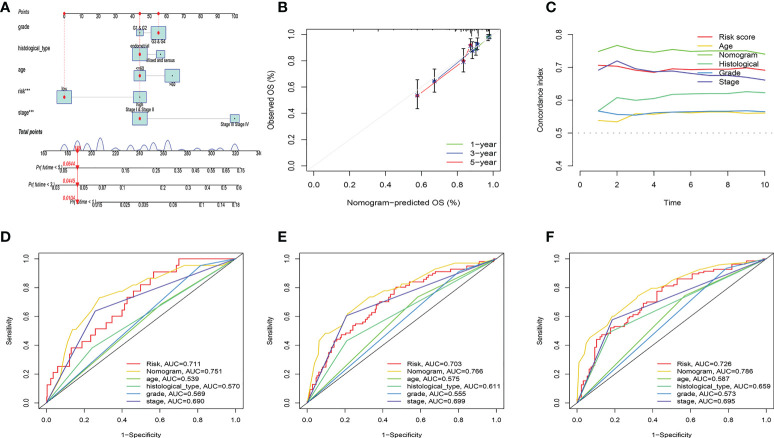
Nomogram construction and evaluation. **(A)** According to the nomogram, the 1-, 3-, and 5-year mortality rates for the patients were 0.0104, 0.0445, and 0.0644, respectively. **(B)** Calibration curve for nomogram. **(C)** C-index curve. **(D-F)** ROC curves.

We built a nomogram by combining riskscore and clinical factors. According to the nomogram, the 1-, 3-, and 5-year mortality rates for the patients were 0.0104, 0.0445, and 0.0644, respectively (**Figure 9A**). The calibration curve showed the nomogram had an excellent calibration (**Figure 9B**). The C-index showed that the nomogram performed better than the risk score and clinical factors (**Figure 9C**). The same conclusion was drawn from the ROC curve, with the AUC of 0.751, 0766, and 0.786 in the nomogram at years 1, 3, and 5, respectively (**Figures 9D–F**).

### Validating gene expression level of cellular senescence-related genes in UCEC samples

To validate the expression levels of cellular senescence-related genes, we used qRT-PCR to detect the expression levels of six cellular senescence-related genes in 12 UCEC samples and 12 normal tissues. The results indicated that ARHGAP29 expression was significantly higher in tumor samples, while GNLY and NRIP1expression was significantly lower in UCEC samples. There was no significant difference in BATF, BZW2 and SIX1 expression (**Figure S6**).

## Discussion

In this study, to evaluate the involvement of senescence genes in UCEC, we did a complete bioinformatics analysis. Based on the senescence gene, we began by categorizing the patients into two groups. Subtype B has a worse prognosis. TME, immune checkpoint gene expression, and immune function also differed significantly between the two subtypes. We further discovered two gene subtypes based on the differential genes. The results of correlation analysis showed that different genes can be used as indicators of patient prognosis and TME. Therefore, the prognostic model was built using differential genes. The model’s predictive ability was proven using survival analysis and ROC curves. Furthermore, this prognostic model was significantly associated with clinical factors, TME, immune-related markers, TMB, MSI, and drug sensitivity. Finally, we built a nomogram by combining riskscore and clinical factors. The results showed that the nomogram was successful in stratifying patients and guiding them in prognostic assessment and treatment selection.

In this study, we verified the expression levels of cell senescence-related genes in tumor tissues and normal tissues. Perhaps due to the small sample size, there was no difference in BATF, BZW2 and SIX1 expression between tumor and normal tissues. It is necessary to expand the sample size to further verify this result. Senescence is a steady state that removes sick cells and stabilizes the collective internal environment (11). It is also thought to prevent tumor development (28). However, recent studies have found that tumor progression can also be caused by cellular senescence (29). Senescent cells secrete signaling molecules that affect tumor proliferation, invasion and metastasis, and angiogenesis (30). In addition, the senescence of some tumor cells is reversible and they can escape cellular senescence and re-enter the cell cycle, which is an important cause of tumor recurrence and progression (31). As a result, it is critical to thoroughly investigate the clinical importance of cellular senescence in malignancies. However, there are currently no studies on the role of cellular senescence in UCEC.

Four genes have been identified as being involved in illness development and progression. BZW2 is a protein that has a role in cell adhesion (32). Huang et al. showed that BZW2 promoted colorectal cancer progression (33). NRIP1 is a nuclear receptor protein, and Its high expression is linked to a bad prognosis of gastric cancer (34). ARHGAP29 is a GTPase that stimulates prostate cancer development and metastasis (35). SIX1 is a transcription factor with an important role in tumorigenesis (36, 37). Our prognostic model combines these four genes, which will give us a better understanding for cancer cells.

The function of programmed cell death in tumor therapy and TME are receiving increasing attention (38, 39). Tumor growth must evade tumor immunity, which is also considered an important marker of tumor progression (40, 41). Despite breakthroughs in the treatment of aggressive malignancies with immunotherapy, a large minority of patients still have no impact on treatment (42, 43). The immune microenvironment of UCEC can predict patient survival (44). In this study, GSEA analysis revealed that the low-risk group was mostly associated to immunity. In addition, our study also found the riskscore was inversely related to the patient’s stromalscore, immunescore, estimatescore and proportional to tumorpurity. At the same time, we also found that major immune checkpoint genes were up-regulated in the low-risk group. This means that patients with low-risk scores are more immunogenic and may benefit from immunotherapy. Therefore, our study may guide the immunotherapy of UCEC patients.

Studies have shown that immunotherapy is more effective in people with a high TMB (45). Tissue TMB can also predict patient response to immune checkpoint therapy (46). TP53 mutation is an independent marker of poor prognosis (47). There is also evidence that human carcinogens can induce TP53 mutations (48). Our study also reached similar conclusions. The mutation rate of TP53 is substantially greater in the high-risk group than in the low-risk group. This helps us explore the causes of tumorigenesis and the choice of treatment options for patients. Besides, drug resistance of tumors has always been one of the challenges of UCEC treatment (49). It is also difficult to effectively treat advanced cases (50). To this end, our study screened drug candidates for relevanche to prognostic models.

Our study has some limitations. First, our studies are all from public databases. Due to the limited access to public data sets and the limited amount of data, the clinicopathological parameters analyzed in this study were not comprehensive, and there were errors or biases. In the future, we will conduct basic experiments *in vivo* or *in vitro* to confirm our findings. Second, our study was a retrospective study. Future prospective clinical validation is needed.

This is the first prognostic model of UCEC based on cellular senescence genes to our knowledge. Our analyses reveal a broad range of regulatory regulatory mechanisms that facilitate individualized treatment and prognosis prediction in patients.

## Conclusion

We constructed a UCEC prognostic model based on cellular senescence genes and combined with clinical factors to construct nomograms, which showed good predictive performance. Using this model, the prognosis and TME of UCEC patients can be accurately estimated. Furthermore, our findings may lead to new approaches for UCEC treatment.

## Data availability statement

The datasets presented in this study can be found in online repositories. The names of the repository/repositories and accession number(s) can be found in the article/**Supplementary Material**.

## Ethics statement

The studies involving human participants were reviewed and approved by the Ethics Committee of the Nantong Maternal and Child Health Hospital Affiliated to Nantong University. The patients/participants provided their written informed consent to participate in this study.

## Author contributions

JT and JJ conceived the study and participated in the study design and performance. LG and XDW conducted the bioinformatics analysis and manuscript writing. XHW and FW revised the manuscript. All authors contributed and approved the submitted version.

## Acknowledgments

We would like to extend our gratitude to the researchers and study patients for their contributions.

## Conflict of interest

The authors declare that the research was conducted in the absence of any commercial or financial relationships that could be construed as a potential conflict of interest.

## Publisher’s note

All claims expressed in this article are solely those of the authors and do not necessarily represent those of their affiliated organizations, or those of the publisher, the editors and the reviewers. Any product that may be evaluated in this article, or claim that may be made by its manufacturer, is not guaranteed or endorsed by the publisher.

## Supplementary material

Supplementary Figure 1 Cluster analysis. Cluster analysis heatmap of cellular senescence genes (k = 3-9).

Supplementary Figure 2 Cluster analysis. **(A)** Cluster analysis heatmap of differential genes (k = 3-9). **(B)** Dividing patients into two subtypes was optimal. **(C)** Survival analysis. Subtype B has a poorer prognosis. **(D)** There were no differences in clinical factors between the two subtypes.

Supplementary Figure 3 Association of clinical factors with risk score. (A-E) Risk score in patients with different age, grade, fustat, and histological_type.

Supplementary Figure 4 Model evaluation. **(A-C)** Patients were divided into high- and low-risk groups based on the median risk score. **(D-F)** As the risk value increased, the proportion of UCEC patients who died increased. **(G-I)** in the high-risk group, BZW2, ARHGAP29, and SIX1 were highly expressed, whereas, in the low-risk group, NRIP1 was highly expressed.

Supplementary Figure 5 Immunoassay of the model. **(A)** The distribution of immune cells for each sample in the two groups. **(B-F)** The relationship between risk scores and immune cells. T cell regulatory (Tregs) were negatively correlated with risk score, and the remaining cells were all positively correlated with risk score. **(G, H)** The relationship between immunophenotyping and risk score. **(I, J)** Immune checkpoint analysis. There were also significant differences in the expression of immune checkpoint genes between the two groups. **(K-M)** PDCD1LG2 was positively associated with risk scores, CTLA4, and PDCD1 were negatively associated with risk scores.

Supplementary Figure 6 The cellular senescence-related genes expression was investigated by qPT-PCR.
