# Supplementary material for: A prognostic model and immune regulation analysis of uterine corpus endometrial carcinoma based on cellular senescence
Source: Front Oncol. 2022 Dec 8;12:1054564. doi: 10.3389/fonc.2022.1054564 (PMC9775865; doi:10.3389/fonc.2022.1054564)
Supplement: Supplementary file 1 [file DataSheet_1.docx]

Supplementary material

**Tables**

Table S1: Univariate COX analysis

| Cohort | ID | HR | P value |
| --- | --- | --- | --- |
| Train |  |  |  |
|  | age | 2.042 | 0.052 |
|  | histological_type | 3.448 | 0.000^*^ |
|  | grade | 2.268 | 0.173 |
|  | stage | 5.565 | 0.000^*^ |
|  | riskScore | 1.514 | 0.000^*^ |
| Test |  |  |  |
|  | age | 1.511 | 0.195 |
|  | histological_type | 2.679 | 0.000^*^ |
|  | grade | 4.329 | 0.014^*^ |
|  | stage | 3.880 | 0.000^*^ |
|  | riskScore | 1.390 | 0.000^*^ |
| Validation |  |  |  |
|  | age | 1.778 | 0.016^*^ |
|  | histological_type | 3.044 | 0.000^*^ |
|  | grade | 3.363 | 0.004^*^ |
|  | stage | 4.116 | 0.000^*^ |
|  | riskScore | 1.458 | 0.000^*^ |

^*^: p < 0.05.

Table S2: Multivariate COX analysis

| Cohort | ID | HR | P value |
| --- | --- | --- | --- |
| Train |  |  |  |
|  | histological_type | 1.907 | 0.062 |
|  | stage | 3.750 | 0.000^*^ |
|  | riskScore | 1.317 | 0.008^*^ |
| Test |  |  |  |
|  | histological_type | 1.569 | 0.155 |
|  | grade | 2.008 | 0.269 |
|  | stage | 3.036 | 0.000^*^ |
|  | riskScore | 1.286 | 0.022^*^ |
| Validation |  |  |  |
|  | age | 1.599 | 0.064 |
|  | histological_type | 1.503 | 0.094 |
|  | grade | 1.374 | 0.484 |
|  | stage | 3.250 | 0.000^*^ |
|  | riskScore | 1.324 | 0.000^*^ |

^*^: p < 0.05.

Table S3 The sequences of cellular senescence-related genes.

| genes | sequences |
| --- | --- |
| BZW2 | TTTCTGGACTCTACAGGCTCAA |
|  | ACCATCATCTATGCGCGTTCC |
| NRIP1 | GGATCAGGTACTGCCGTTGAC |
|  | CTGGACCATTACTTTGACAGGTG |
| ARHGAP29 | CCTTATGGGAGATGTAGGCAATG |
|  | AGCTCGATAGAGTCAGTGTTCT |
| SIX1 | CTGCCGTCGTTTGGCTTTAC |
|  | GCTCTCGTTCTTGTGCAGGT |
| BATF | TATTGCCGCCCAGAAGAGC |
|  | GCTTGATCTCCTTGCGTAGAG |
| GNLY | CCTGTCTGACGATAGTCCAAAAA |
|  | GACCTCCCCGTCCTACACA |
| BZW2 | TTTCTGGACTCTACAGGCTCAA |
|  | ACCATCATCTATGCGCGTTCC |
