# Supplementary figures and images for: A prognostic model and immune regulation analysis of uterine corpus endometrial carcinoma based on cellular senescence
Source: Front Oncol. 2022 Dec 8;12:1054564. doi: 10.3389/fonc.2022.1054564 (PMC9775865; doi:10.3389/fonc.2022.1054564)

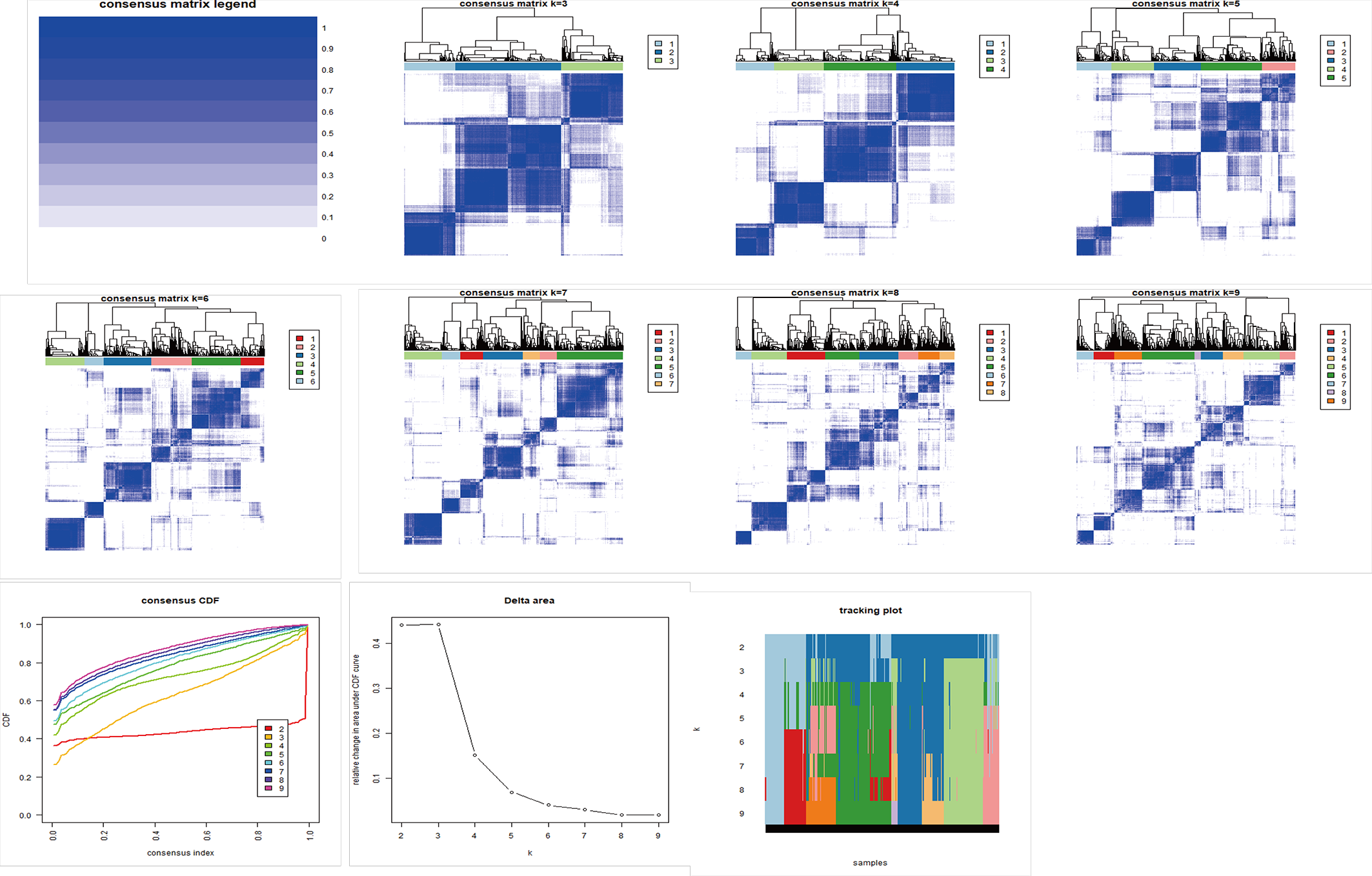

Supplement: Supplementary Figure 1 — Cluster analysis. Cluster analysis heatmap of cellular senescence genes (k = 3-9). [file Image_1.tif]

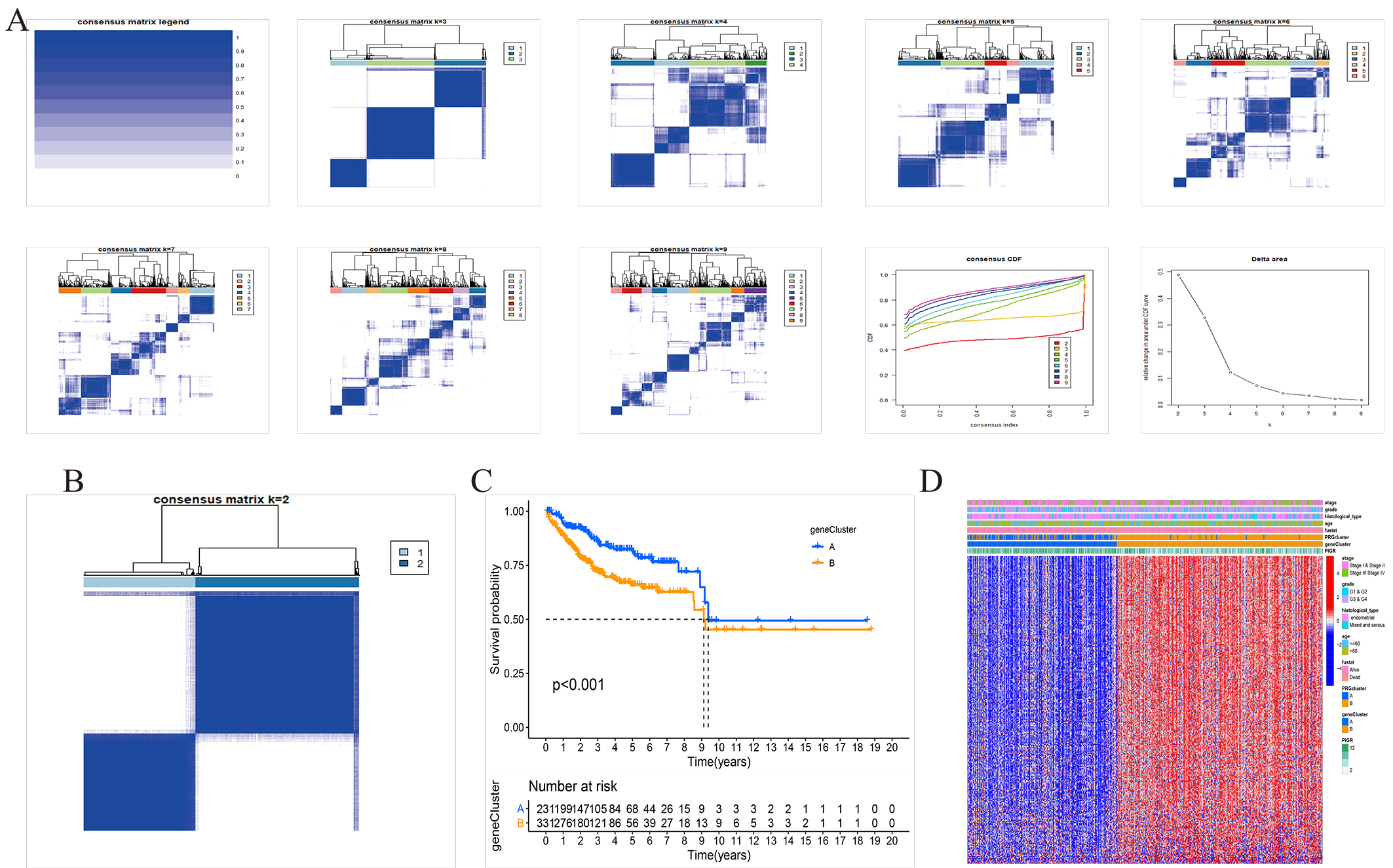

Supplement: Supplementary Figure 2 — Cluster analysis. (A) Cluster analysis heatmap of differential genes (k = 3-9). (B) Dividing patients into two subtypes was optimal. (C) Survival analysis. Subtype B has a poorer prognosis. (D) There were no differences in clinical factors between the two subtypes. [file Image_2.tif]

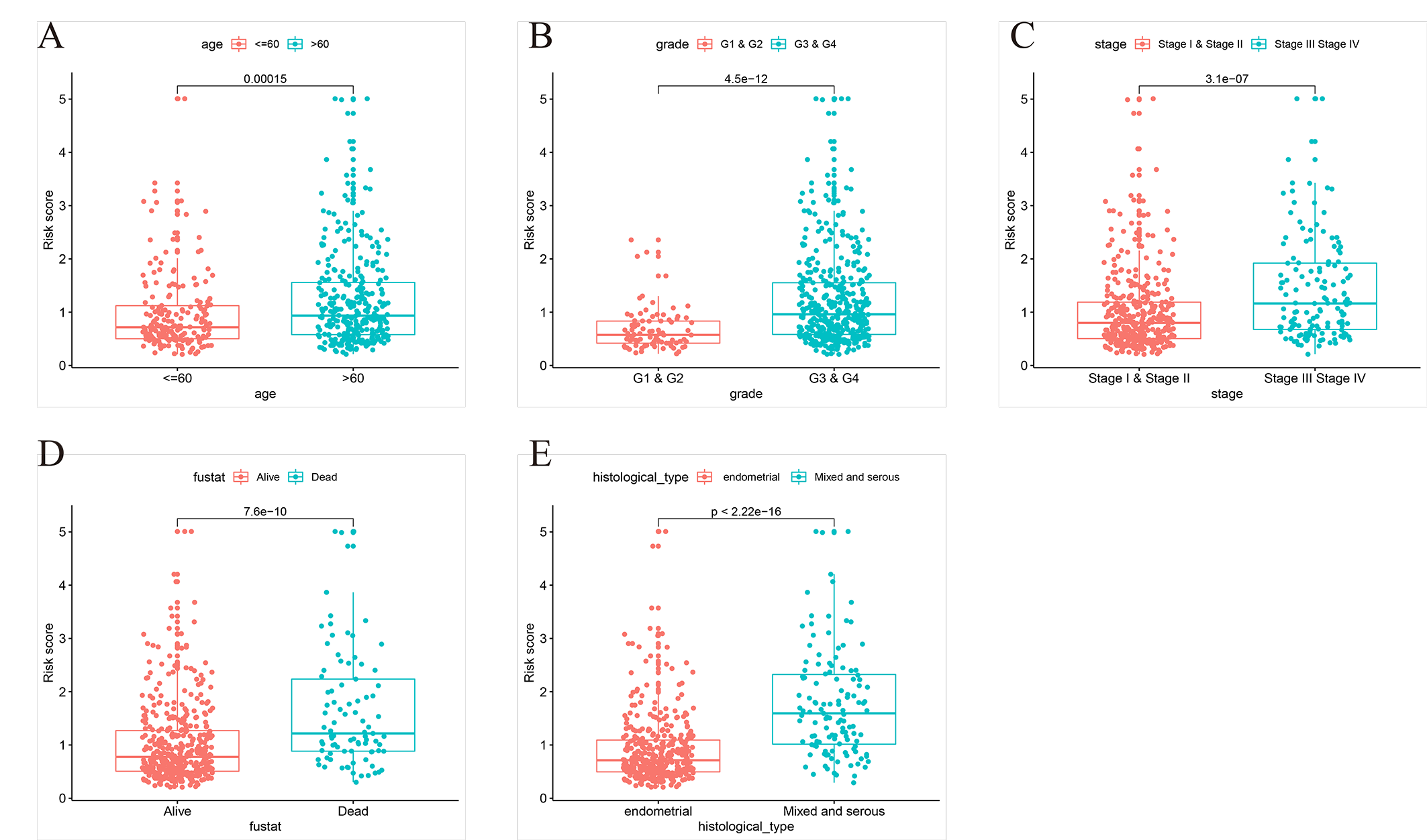

Supplement: Supplementary Figure 3 — Association of clinical factors with risk score. (A-E) Risk score in patients with different age, grade, fustat, and histological_type. [file Image_3.tif]

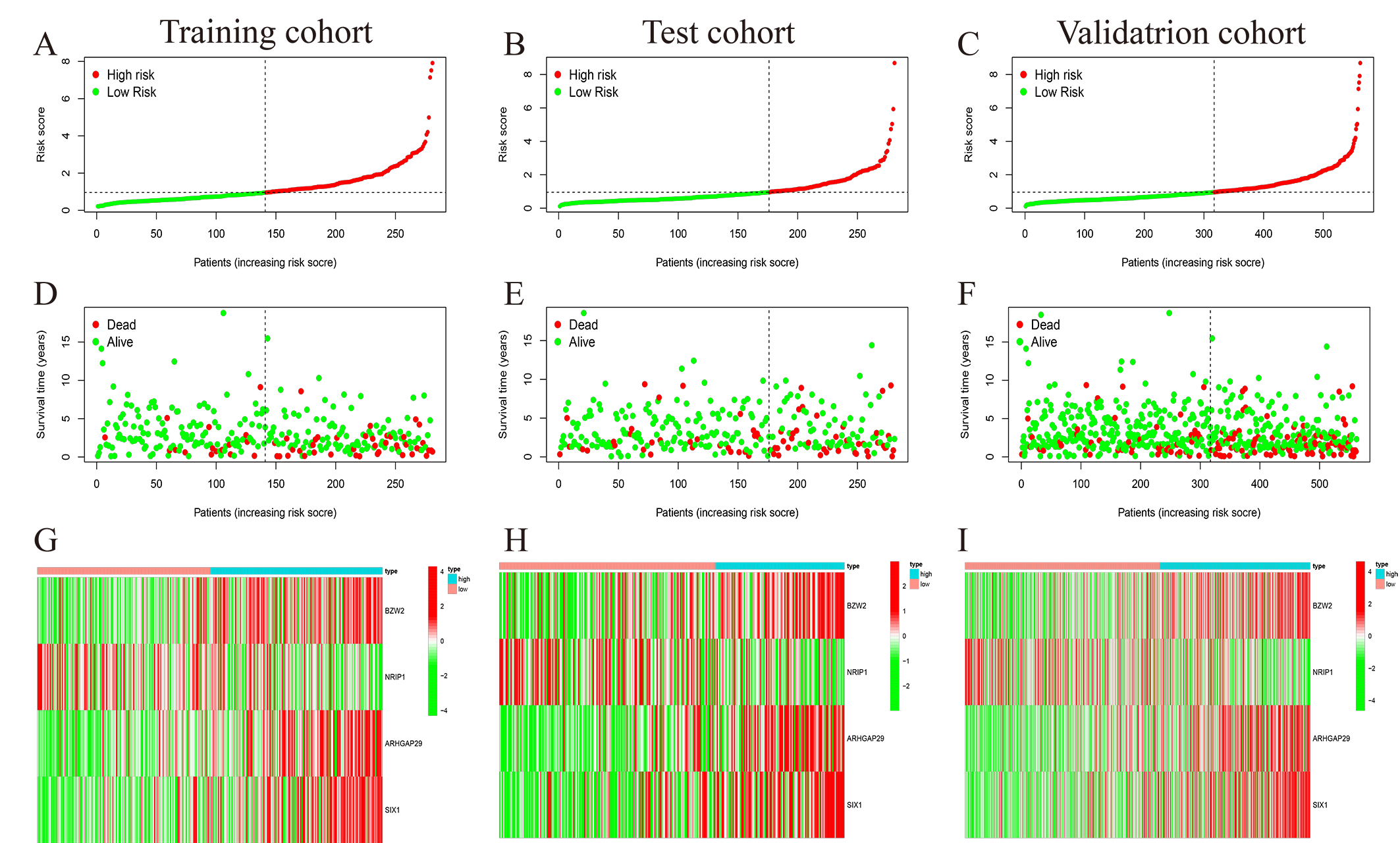

Supplement: Supplementary Figure 4 — Model evaluation. (A-C) Patients were divided into high- and low-risk groups based on the median risk score. (D-F) As the risk value increased, the proportion of UCEC patients who died increased. (G-I) in the high-risk group, BZW2, ARHGAP29, and SIX1 were highly expressed, whereas, in the low-risk group, NRIP1 was highly expressed. [file Image_4.tif]

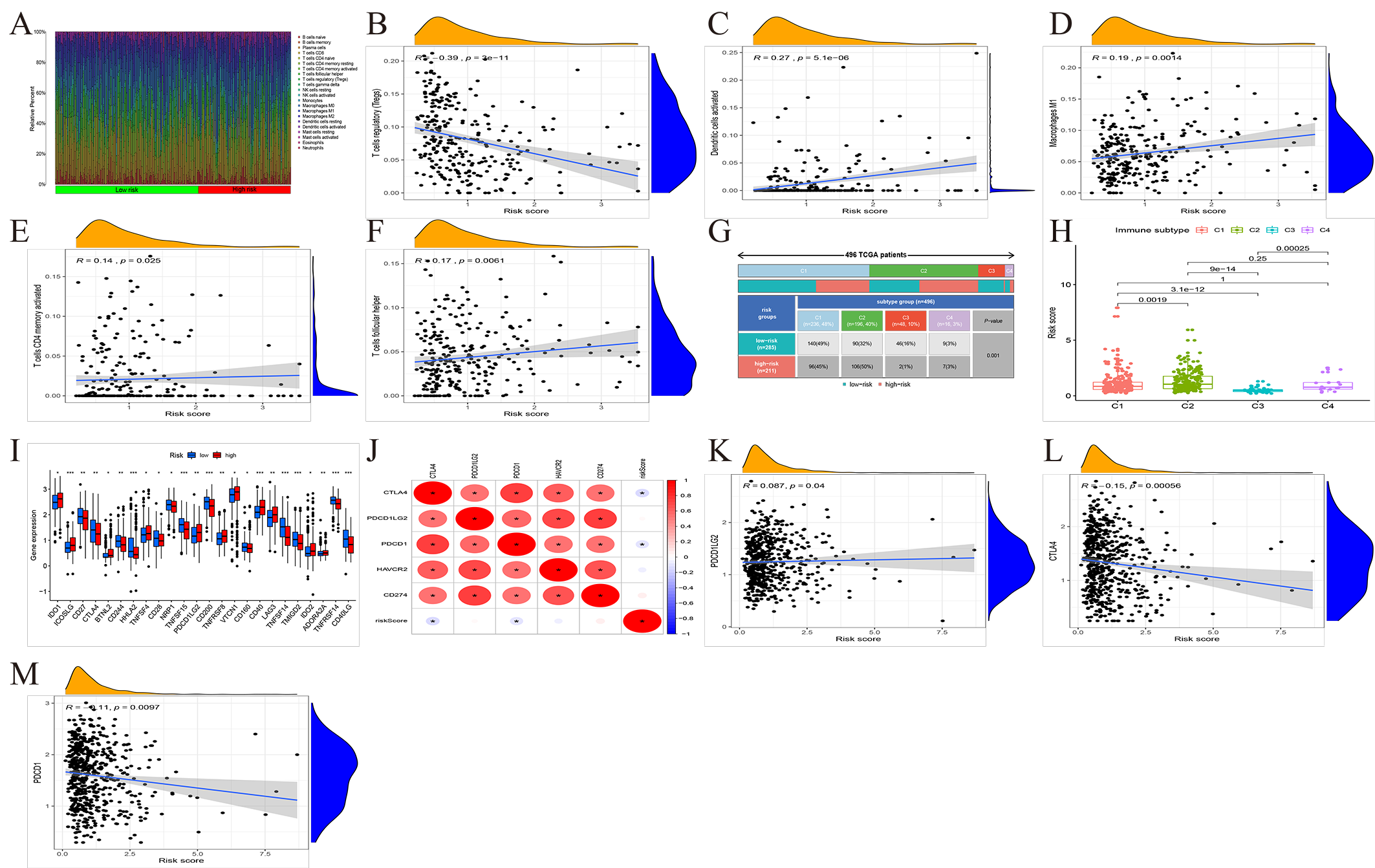

Supplement: Supplementary Figure 5 — Immunoassay of the model. (A) The distribution of immune cells for each sample in the two groups. (B-F) The relationship between risk scores and immune cells. T cell regulatory (Tregs) were negatively correlated with risk score, and the remaining cells were all positively correlated with risk score. (G, H) The relationship between immunophenotyping and risk score. (I, J) Immune checkpoint analysis. There were also significant differences in the expression of immune checkpoint genes between the two groups. (K-M) PDCD1LG2 was positively associated with risk scores, CTLA4, and PDCD1 were negatively associated with risk scores. [file Image_5.tif]

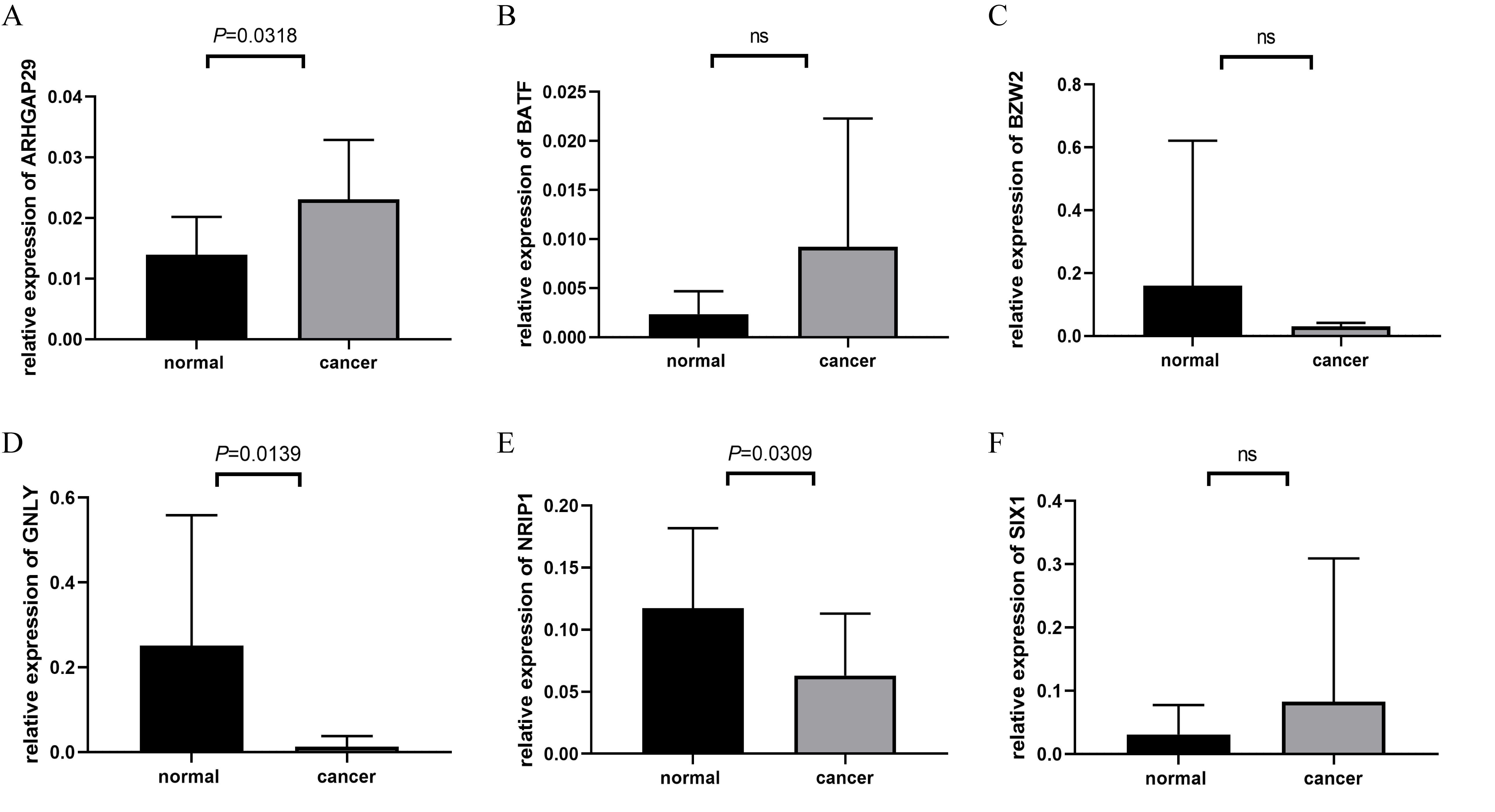

Supplement: Supplementary Figure 6 — The cellular senescence-related genes expression was investigated by qPT-PCR. [file Image_6.tif]
